# ATF4 promotes brain vascular smooth muscle cells proliferation, invasion and migration by targeting miR-552-SKI axis

**DOI:** 10.1371/journal.pone.0270880

**Published:** 2022-07-20

**Authors:** Meina Feng, Qin Zhou, Wenxian Tu, Yunfeng Wang, Yuanmin Du, Kang Xu

**Affiliations:** Department of Neurology, Wuhan Brain Hospital, General Hospital of the YANGTZE River Shipping, Wuhan, China; University of California San Francisco, UNITED STATES

## Abstract

**Background:**

Studies have indicated vascular smooth muscle cells (VSMCs) played a crucial role in atherosclerosis and microRNAs (miRNAs) played key roles in biological functions of VSMCs. Whereas, the potential function and mechanism of miR-552 in VSMCs remains unclear. Our aim was to explore the role of miR-552 on VSMCs and underlying mechanism.

**Material/Methods:**

MTT assay and transwell assay were used to measure the proliferation, invasion, and migration of human brain VSMCs (HBVSMCs) and mice VSMCs (mVSMCs), respectively. Bioinformatics tools and luciferase assay were adopted to verify the association between miR-552 and SKI. Rescue experiments were employed to assess the interaction of miR-552 and SKI in modulating biological functions in HBVSMCs and mVSMCs. The expression level of transcription factors (TFs)was measured via qRT-PCR assay. The effect of ATF4 on miR-552 and SKI expression was tested by qRT-PCR or western blot assay. Finally, chromatin immunoprecipitation (ChIP) assay and JASPAR databases were used to analyze the regulatory linkage between ATF4 and miR-552.

**Results:**

We found that miR-552 was upregulated in HBVSMCs treated with PDGF-bb and miR-552 overexpression could promote proliferation, invasion, and migration of HBVSMCs and mVSMCs, whereas, miR-552 knockdown had the opposite impact. In addition, we also found that SKI was a direct target of miR-552, which reversed miR-552-mediated proliferation, invasion, and migration in HBVSMCs and mVSMCs. Furthermore, we also discovered that miR-552 overexpression promoted the effects of ATF4 elevation on proliferation, migration and invasion of HBVSMCs and mVSMCs, but, miR-552 decline had the opposite impact.

**Conclusions:**

ATF4-miR-552-SKI axis played critical roles in the proliferation and migration of HBVSMCs and mVSMCs, which were closely involved in atherosclerosis (AS). Therefore, our findings might offer a novel therapeutic target for AS.

## Introduction

Atherosclerosis (AS), a chronic and devastating disease featured by the aggregation of fibrous ingredients and lipids in the wide arteries, which results in most of the high morbidity and mortality worldwide [1]. Various evidence demonstrated that vascular smooth muscle cells (VSMCs) had a critical role in the progression of atherosclerosis [2]. The abnormal proliferation of VSMCs and the phenotypic modulation from contractile to the secreting phenotype are regarded to promote the vascular remodeling [3,4]. Thus, the potential molecular mechanism in VSMCs proliferation is worthy of exploring to find the new effective therapy for AS.

MiRNAs are a group of highly conserved, short and functional non-coding RNAs, which are associated with regulating protein translation through targeting the target mRNA [5]. Increasing studies reported that miRNAs were involved in numerous biological processes, such as vascular remodeling, inflammation as well as oxidative stress, which had a significant effect on the progression of AS [6,7]. For instance, Sun et al. indicated that miR-146b-5p protected against atherosclerosis via inhibiting VSMC proliferation and migration [8]. Guo et al. found that hsa-miRNA-23a-3p promoted atherogenesis in a novel mouse model of atherosclerosis [9]. Wang et al. identified that miR-761 modulated foam cell formation and inflammation through autophagy in the progression of atherosclerosis [10]. In addition, several studies indicated that miR-552 played critical roles in cancer development [11–13]. Whereas, little is known about the role of miR-552 in HBVSMC proliferation, migration and invasion. SKI, one of the miR-552 target genes identified in the present study, has been reported to play a protective role in VSMC against atherosclerosis [14]. Exploration the role of miR552/SKI axis in VSMCs proliferation may have broad prospects for clinical combined intervention of atherosclerosis.

In the present study, we found that miR-552 could promote the proliferation, migration and invasion of human VSMCs (HBVSMCs) and mouse VSMCs (mVSMCs) by targeting SKI. In addition, in order to unveil the upstream regulatory factor of miR-552, transcription factors (TFs) that regulated promoters of miR-552 was predicted based on JASPAR. This study discovered that the ATF4-miR-552-SKI axis played a key role in VSMC development and could be a novel target for therapy of cerebro-cardiovascular atherosclerosis.

## Methods and materials

### Cell culture and transfection

The HBVSMCs were purchased from American Type Culture Collection (ATCC, Manassas, VA, USA) and were cultured in DMEM containing 10% fetal bovine serum (FBS), 1% penicillin and 1% streptomycin (Gibco) in a humidified incubator at 37°C and in 5% CO_2_.

The mVSMCs were isolated from the aorta (from the aortic arch to the iliac bifurication) of C57BL/6J aged at 6 weeks. Briefly, the aortic intima was collected and minced with a scissor. The resulting cells were seeded in six-well culture plates in DMEM supplemented with 20% FBS, 1% penicillin and 1% streptomycin at 37C, 5% CO_2_ atmosphere. Cells at passages 3–5 were used in downstream experiments.

The miR-552 mimics, miR-552 inhibitors, scramble, were obtained from GenePharma (Shanghai, China). The pcDNA3.1 (Vector) and pcDNA3.1- SKI overexpression (SKI-OE) plasmids were provided by GenePharma (Shanghai, China). The pcDNA3.1 (Vector) and pcDNA3.1-ATF4 overexpression (ATF4-OE) plasmids were constructed by GenePharma (Shanghai, China). The transfection of HBVSMCs was performed using Lipofectamine 2000 (Invitrogen, USA) in accordance to the manufacturer’s instructions. After transfection for 48h, the target cells were harvested for further analysis.

### Western blot

Total cells were lysed by using RIPA lysis buffer (Beyotime Institute of Biotechnology) and then concentrations of proteins were detected with the Pierce BCA Protein Assay kit (Thermo Scientific, Belgium) in accordance to the manufacturer’s protocols. Next, 30 μg proteins were separated through SDS-PAGE and then transferred to PVDF membrane (Millipore, USA). After being blocked with 5% milk in TBST buffer (Thermo Scientific, USA), the membrane was incubated by specific primary antibodies (GAPDH, 1:10000, ab181602, Abcam; SKI, 1:1500, ab192520, Abcam; ATF4, 1:1000, ab184909, Abcam; MCM2, 1:1000, ab108935, Abcam; PCNA, 1:2500, ab29, Abcam) overnight at 4°C. Then, the membrane was incubated with secondary antibody at room temperature for 1 h. Finally, chemiluminescent detection was carried out with hypersensitive ECL (Biossci Biotechnology Co, Ltd., Wuhan, China).

## Quantitative reverse transcription-polymerase chain reaction (qRT-PCR)

Total RNA was isolated from HBVSMCs or mVSMCs via TRIzol reagent (Invitrogen, Carlsbad, USA) based on the manufacturer’s protocol. After that, the RNA was reversely transcribed into cDNA by PrimeScript-RT Kit (Takara, Kusatsu, Japan). RT-qPCR was conducted in accordance to the instructions of SYBR Premix Ex Taq™ II (Takara). The primers employed in this study were presented in **S1 Table**. GAPDH and U6 were adopted as internal controls and the relative expression was calculated according to the 2^–ΔΔCt^ method.

### Cell proliferation assay

The proliferation of HBVSMCs and mVSMCs was assessed using MTT assay (Sigma, USA). Briefly, 5×10^3^ transfected HBVSMCs were seeded in 96-well plates at 37°C, followed by addition of 20 μL MTT solution, which was then dissolved via 150μl DMSO (Sigma). The optical density (OD) value was measured at 490 nm through the microplate reader.

### Transwell assay

Transwell was performed to assess the mobility of HBVSMCs and mVSMCs. Briefly, the transfected VSMCs(4×10^4^ cells/well) were seeded in the upper chamber in serum-free medium and the DMEM supplemented with 10% FBS was pipetted into the lower chamber. After post-incubation for 24 hours, the migrated cells were fixed with 4% paraformaldehyde and stained with crystal violet. Finally, the migrated cell number was assessed by a microscope.

### Luciferase reporter assay

To unveil the binding correlation between SKI 3’UTR and miR-552, PMIR-REPORT luciferase vector (GenePharma, Shanghai, China) with wild-type-SKI-3’UTR (Wt-SKI-3’UTR) or mutant (Mut)-SKI-3’UTR, miR-552 mimic or miR-552 knockdown (miR-552-KD) or miR-NC was transfected into HBVSMCs. After culture for 48h, the luciferase activities were tested using a dual-luciferase reporter assay system (Promega) in accordance to the manufacturer’s protocols. All experiments were conducted in triplicate.

### Chromatin Immunoprecipitation (ChIP) assay

ChIP assay was conducted with a ChIP kit (Abcam) in accordance to the manufacturer’s protocols. Briefly, transfected VSMCs (2×10^5^ cells per well) were seeded in 24‐well plates and cultured for 24 hours. On the next day, formaldehyde cross-linked chromatin was sonicated into 200- to 1000-bp fragments under the proper conditions, followed by the incubation of the primary antibody overnight at 4°C. The DNA fragments containing the DNA-binding domain of ATF4 with miR-552 were further confirmed by qRT-PCR based on the specific primers.

### Statistical analysis

All statistical analyses were conducted by GraphPad Prism 6. Two-tailed Student’s t-test was adopted to evaluate significant differences between two groups. One-way ANOVA was employed to assess three or more groups. P value less than 0.05 were considered to be statistically significant.

## Results

### MiR-552 was increased in PDGF-bb-induced HBVSMCs

**Fig 1:**
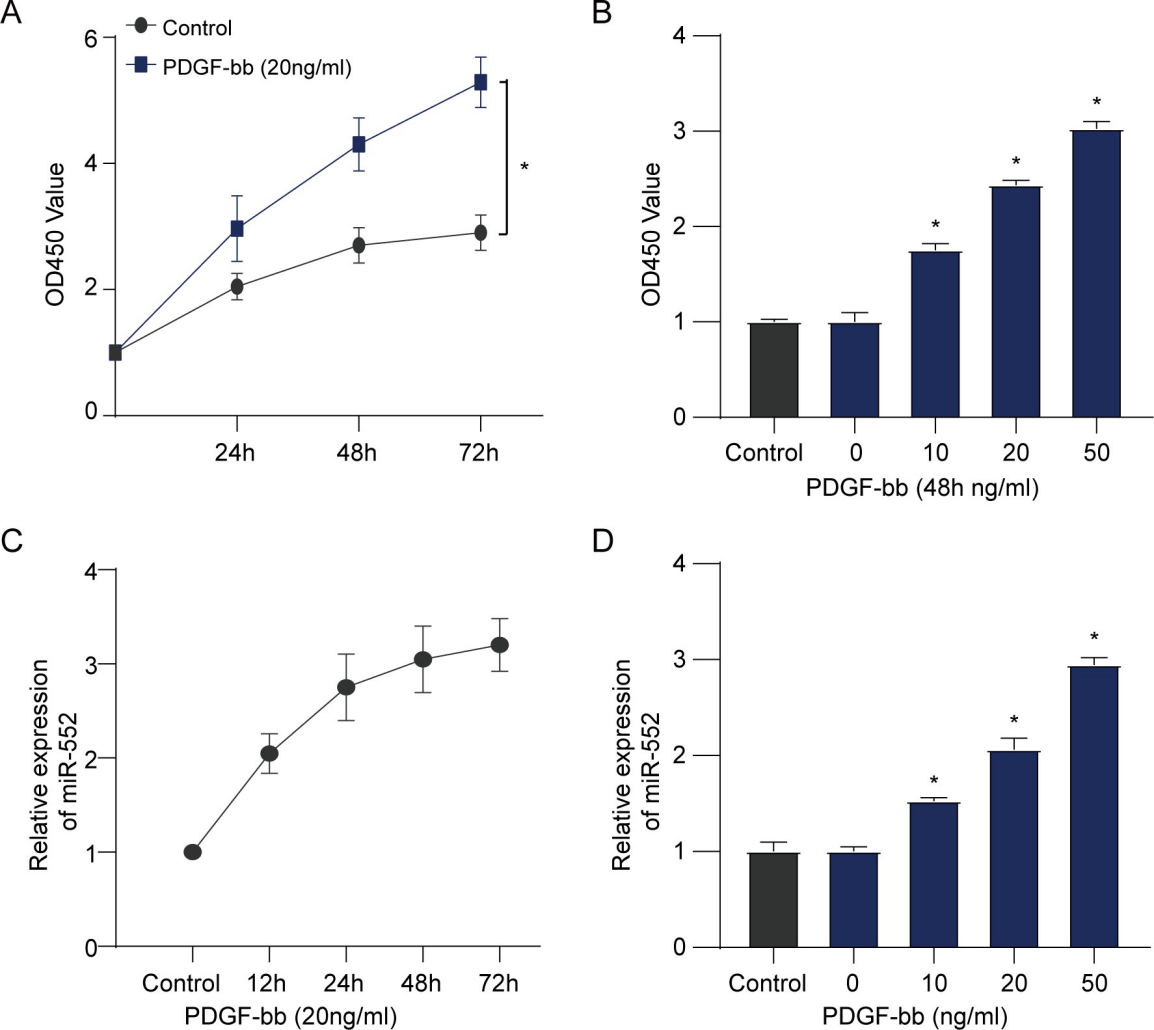
PDGF-bb enhanced the cell viability of HBVSMCs and promoted miR-552 expression. **(A, C)** MTT assay was used to assess the cell viability and qRT-PCR assay was used to assess miR-552 expression of HBVSMCs at different time points following treatment using 20 ng/mL PDGF-bb. (Student’s t test). **(B, D)** MTT assay was used to evaluate the cell viability and qRT-PCR assay was used to measure miR-552 expression of HBVSMCs at 48 h after treatment by PDGF-bb at different doses. (Student’s t test). *P < 0.05. ns: No significance.

MTT assay was adopted to assess the effect of PDGF-bb on the proliferation viability of HBVSMCs, and qRT-PCR assay was conducted to assess the expression level of miR-552. The result of HBVSMCs viability indicated that PDGF-bb enhanced the proliferation of HBVSMCs according to a time-dependent and dose-dependent method (**Fig 1A and 1B**). In addition, we found that PDGF-bb could promote the expression of miR-552 in HBVSMCs in a time- and dose-dependent manner (**Fig 1C and 1D**).

### MiR-552 facilitates proliferation, invasion and migration of VSMCs

**Fig 2:**
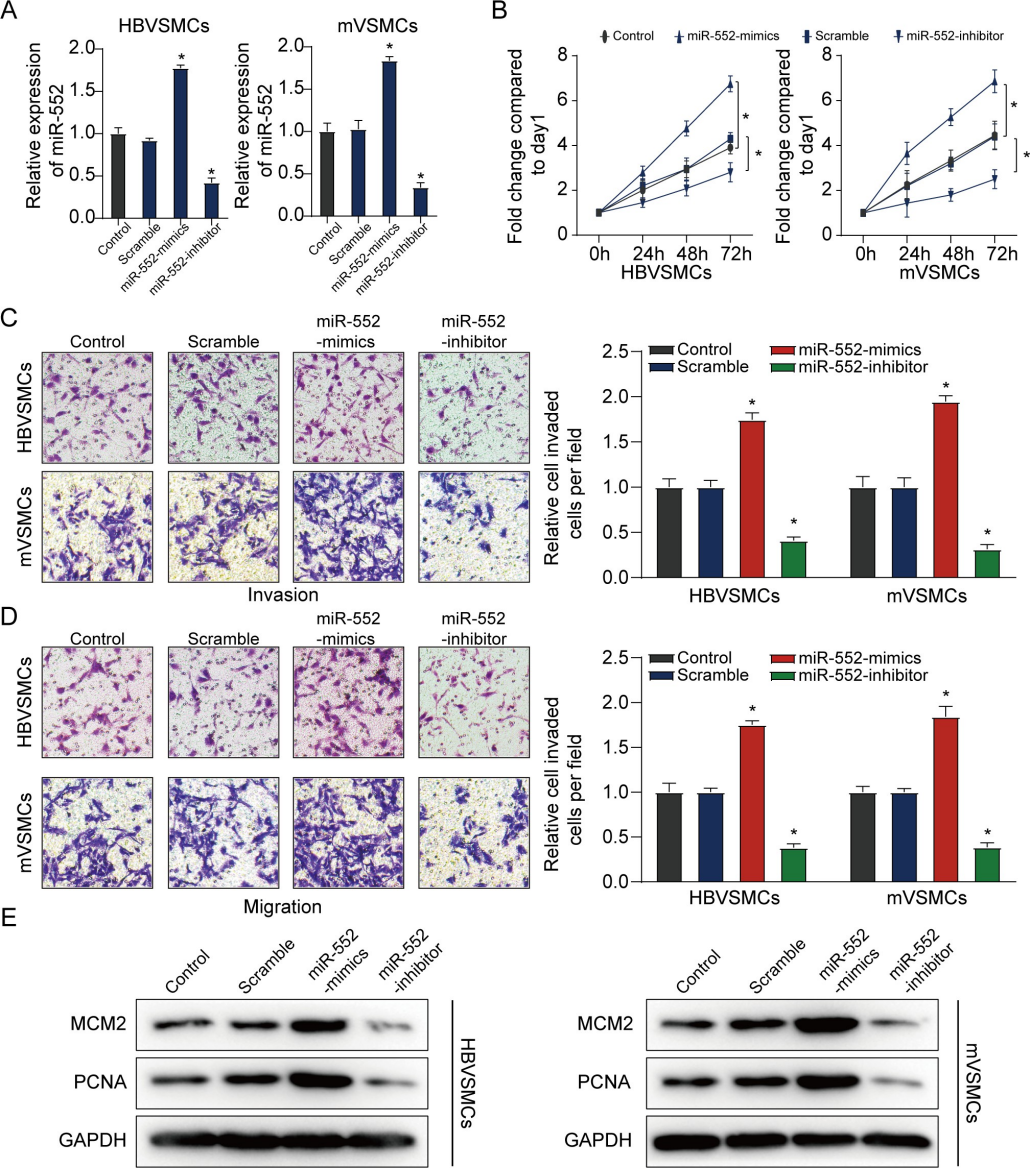
MiR-552 facilitates proliferation, invasion and migration of HBVSMCs. **(A**) qRT-PCR assay was used to measure miR-552 expression in HBVSMCs and mVSMCs transfected using miR-552 mimics or inhibitor. (**B**) The MTT assay was applied to assess proliferation ability of HBVSMCs and mVSMCs transfected by miR-552 mimics, inhibitor, scramble or control. (**C, D**) The transwell assay was conducted to measure invasion and migration of HBVSMCs and mVSMCs transfected with miR-552 mimics, inhibitor, scramble or control, respectively. **(E)** Western blot assay was used to detect the protein level of PCNA and MCM2 in HBVSMCs and mVSMCs transfected with miR-552 mimics, inhibitor, scramble or control, respectively. (Student’s t test). *P < 0.05. ns: No significance.

To further explore the potential molecular mechanism of miR-552 in HBVSMCs and mVSMCs proliferation, we overexpressed miR-552 via miR-552 mimics and reduced miR-552 expression via its inhibitor (**Fig 2A**). As expected, overexpression of miR-552 could clearly promote the proliferation, invasion as well as migration of HBVSMCs and mVSMCs in comparison with controls. But decline of miR-552 significantly blocked the proliferation, invasion and migration of HBVSMCs and mVSMCs compared with the scramble group and the untreated group (**Fig 2B–2D**). Additionally, we found that miR-552 overexpression promoted the expression levels of proliferation-associated genes including PCNA and MCM2 in HBVSMCs and mVSMCs, which was clearly diminished by miR-552 inhibitor (**Fig 2E**), suggesting that miR-552 had a promoting effect on cell growth and migration of HBVSMCs and mVSMCs.

### MiR-552 targets SKI in VSMCs

**Fig 3:**
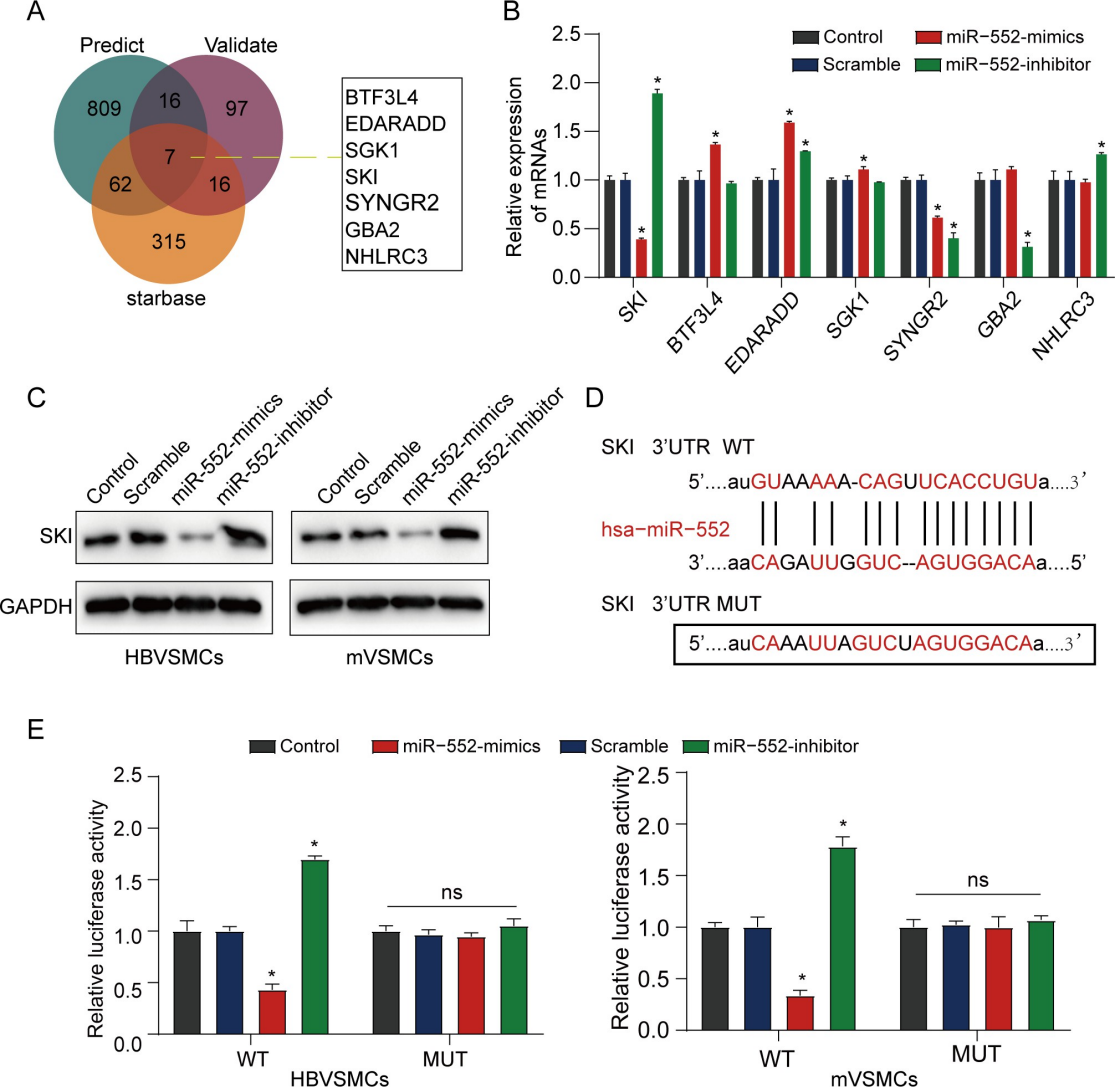
MiR-552 targets SKI in HBVSMCs. **(A)** Venn diagram among miRWalk predicted, miRWalk validated and Starbase sets. (**B**) qRT-PCR analysis for the determined seven DEGs in HBVSMCs treated with PDGF-bb. (**C**) Western blot assay was used to detect the protein level of SKI in VSMCs (HBVSMCs and mVSMCs) when they were transfected with miR-552 mimics, inhibitor, scramble or control, respectively. (**D**) Bioinformatics analysis predicted putative miR-552 target sites of SKI 3’UTR. (**E**) VSMCs (HBVSMCs and mVSMCs) were co-transfected with SKI-WT or SKI-MUT and miR-552 mimics or miR-NC mimics, after 48 h following transfection, luciferase activities were detected. (Student’s t test). *P < 0.05. ns: No significance.

Following this, we analyzed the underlying mechanisms of miR-552 in HBVSMCs. Seven latent common target genes were identified through overlapping analysis among miRWalk predicted, miRWalk validated and starbase database (**Fig 3A**). The result of the qRT-PCR assay indicated that the expression level of SKI was much lower in HBVSMCs treated with PDGF-bb than that in control groups **(Fig 3B)**. A similar result in protein level was obtained in **Fig 3C**. Starbase was used to predict the binding site of miR-552 to SKI. The results indicated that SKI might be an underlying target of miR-552 (**Fig 3D**). In order to validate this hypothesis, HBVSMCs and mVSMCs were co-transfected with the SKI reporter plasmid and miR-552 mimic. The result showed that the luciferase activity in miR-552 mimic and SKI wildtype co-transfected VSMCs (HBVSMCs and mVSMCs) was obviously decreased. whereas, the luciferase intensity in mutant SKI did not change (**Fig 3E**). These data indicated that SKI was a direct target of miR-552.

### SKI reversed the promotional effect of miR-552 on the proliferation and migration of VSMCs

**Fig 4:**
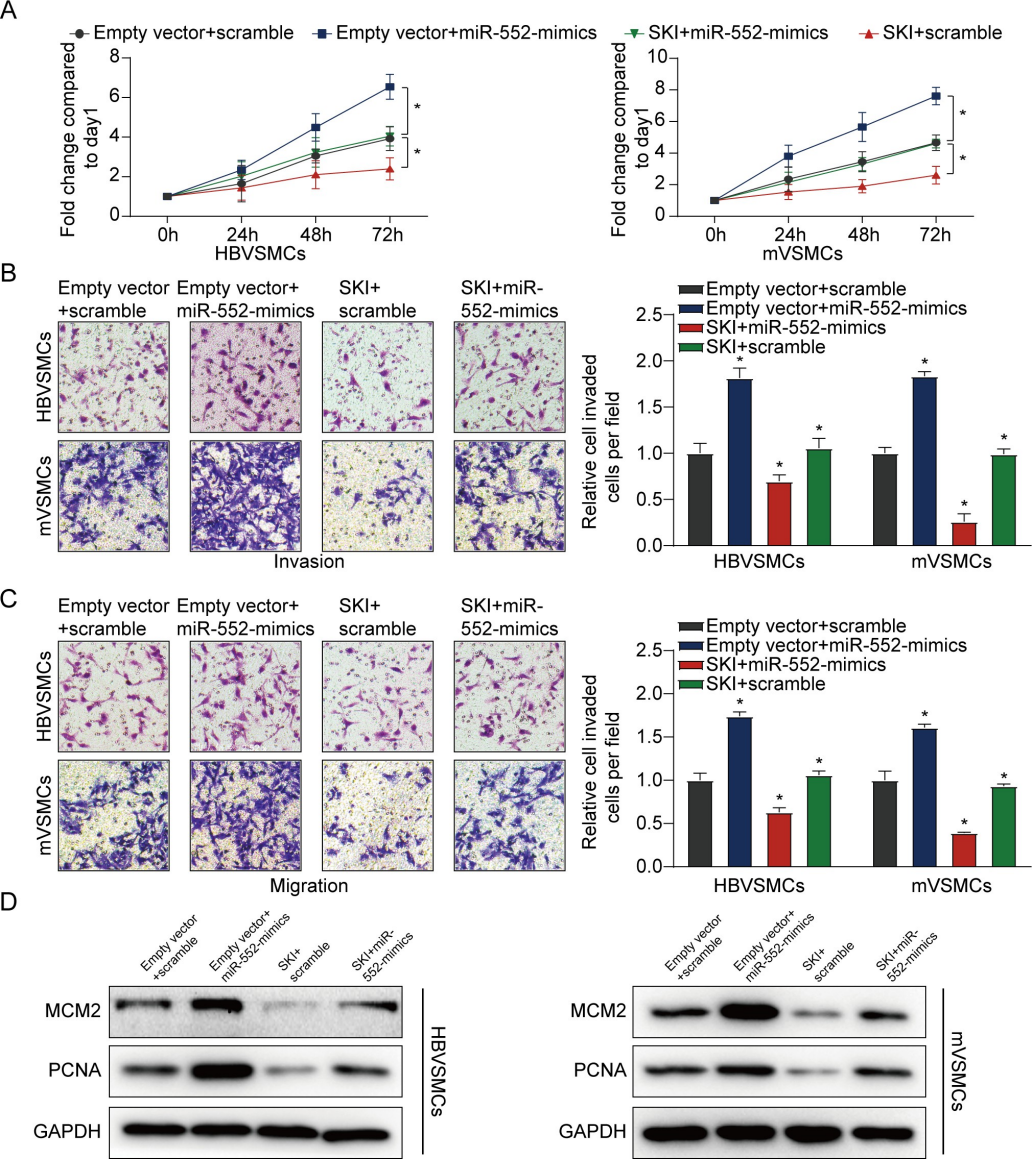
Restoration of SKI reverses the promoting effect of miR-552 on VSMCs. (**A**) The influence of miR-552 and SKI on the proliferation of HBVSMCs and mVSMCs was detected by MTT assay. **(B, C**) The effect of miR-552 and SKI on the invasion and migration of HBVSMCs and mVSMCs was measured according to transwell assay. (**D**) The effect of miR-552 and SKI on the protein level of MCM2 and PCNA in HBVSMCs and mVSMCs was assessed by western blot assay. (LSD post hoc test in conjunction with ANOVA). *P < 0.05. ns: No significance.

We also found that miR-552 overexpression clearly enhanced the proliferation and migration of VSMCs (HBVSMCs and mVSMCs), which could be clearly reversed by high SKI expression (**Fig 4A–4C**). On the other hand, we also discovered that miR-552 overexpression augmented the expression levels of PCNA and MCM2 in VSMCs, which was significantly rescued by SKI upregulation (**Fig 4D**). These collective results suggested that miR-552 inhibited SKI expression by targeting to 3ʹUTR of SKI mRNA in VSMCs.

### ATF4 transcriptionally augmented miR-552 expression

**Fig 5:**
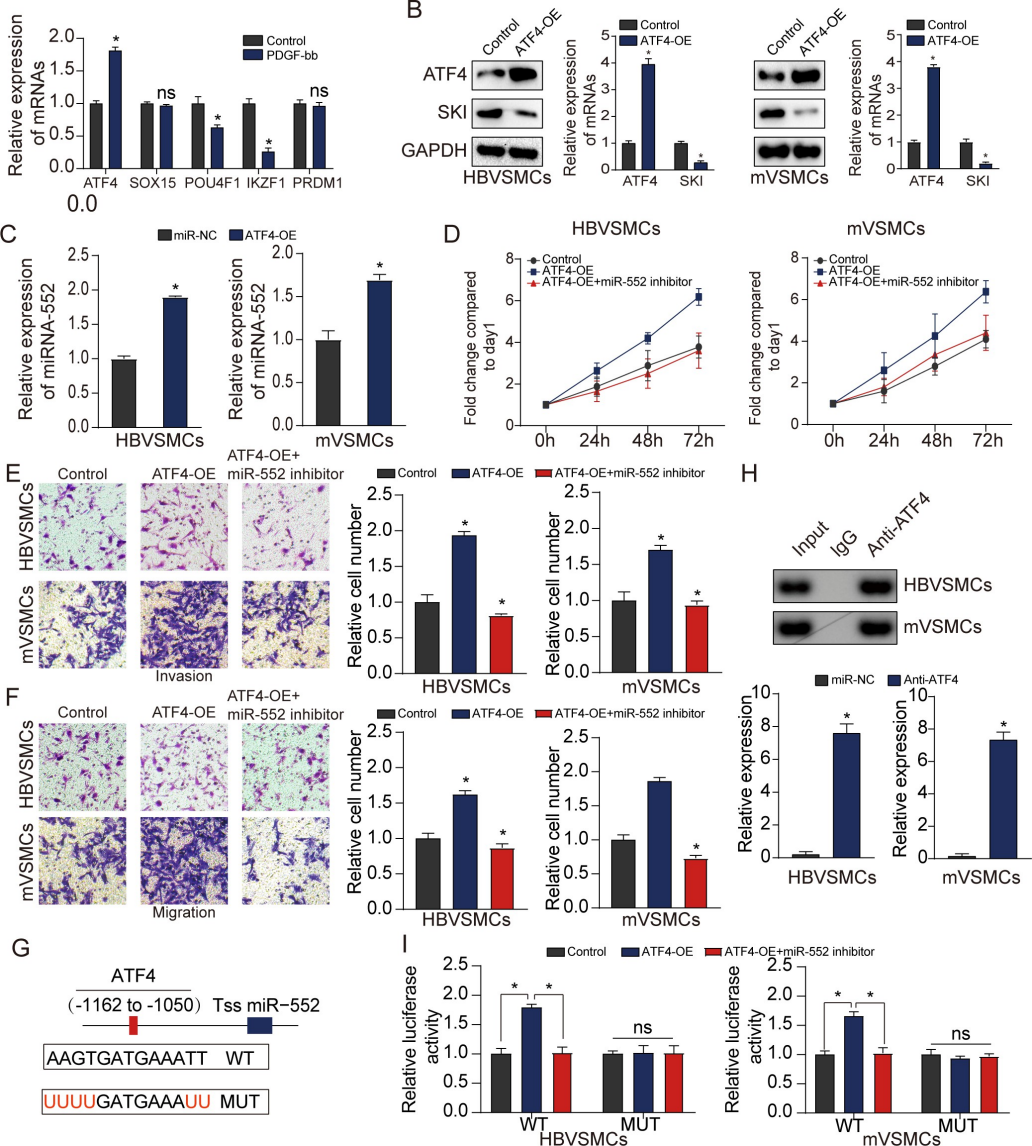
MiR-552 is a target gene of ATF4 in VSMCs. **(A**) qRT-PCR assay was conducted to assess the expression level of the top five TFs expression. (Student’s t test) (**B**) The impact of ATF4 elevation on SKI in HBVSMCs and mVSMCs was tested with western blot assay. (Student’s t test) **(C**) The effect of ATF4 overexpression on miR-552 level in HBVSMCs and mVSMCs was tested on the basis of qRT-PCR assay. (Student’s t test) (**D)** Impact of ATF4 and miR-552 on proliferation of HBVSMCs and mVSMCs in accordance to MTT analysis. (**E, F**) Impact of ATF4 and miR-552 on the invasion and migration of HBVSMCs and mVSMCs was assessed using transwell assay. **(G)** A schematic was used to illustrate the proximal region of the miR-552 promoter based on ChIP assay. (**H)** ChIP assay was applied to manifest the binding association between ATF4 and the miR-552 promoter in HBVSMCs and mVSMCs. (**I**) The luciferase activity in the WT miR-552 promoter was elevated in HBVSMCs and mVSMCs with ATF4 upregulation and this effect could be rescued when they were transfected with miR-552 inhibitor. whereas no change was found in luciferase activity with mutation of ATF4 binding site at -1162 to -1050bp in of the pre-miR-552 promoter region’ upstream. Each experiment was performed in triplicate, (D-I, LSD post hoc test in conjunction with ANOVA). *P < 0.05, ns, no significant.

To explore the upstream regulatory molecule of miR-552, JASPAR software online was used to predict TFs regulating promoters of miR-552. qRT-PCR assay was used to detect the expression change of the top five potential TFs that could transcriptionally regulate the expression of miR-552. Our results demonstrated that the expression level of ATF4 was significantly upregulated in HBVSMCs treated with PDGF-bb compared with that in control group (**Fig 5A**). Additionally, western blot assay was employed to measure the relative expression alteration of SKI with high ATF4 expression. The result illustrated that SKI expression were obviously diminished on the condition of ATF4 upregulation in HBVSMCs and mVSMCs (**Fig 5B**). As demonstrated in **Fig 5C**, the result suggested that ATF4 could elevate miR-552 expression level. We also found that ATF4 upregulation could promote the proliferation, invasion and migration of HBVSMCs and mVSMCs, which could be clearly reversed by miR-552 inhibitor (P<0.05) (**Fig 5D–5F**).

Subsequently, ChIP assay was adopted to further verify the binding of ATF4 to the promoter region of miR-552. Binding sites of ATF4 inside the assumed miR-552 promoter region was predicted using the JASPAR software. In addition, luciferase assays were employed to directly assess the impact of ATF4 on the transcriptional activity of the miR-552 promoter that contained a predicted ARE site. As illustrated in **Fig 5G**, ATF4 could target to the ARE site to activate the transcriptional act of the miR-552 promoter. Additionally, ChIP assay and luciferase reporter assay verified the direct binding of ATF4 to the miR-552 promoter (**Fig 5H and 5I**). All these data suggested that the ATF4-Mir-552-SKI axis plays a key role in promoting the proliferation, invasion and migration of VSMCs, which may may provide a target for clinical atherosclerotic diseases.

## Discussion

In normal arteries, VSMCs are located in the medial layer, responsible for artery contraction and secretion of extracellular matrix (ECM), and play an important role in maintaining arterial compliance and elastic rebound. VSMCs in normal arteries express a series of "SMC markers", usually including smooth muscle cell myosin heavy chain (MYH111), SM22α/TAGLN, smooth muscle cell actin (ACTA2), etc. In atherosclerotic vessels, the expression of these markers of VSMCs is reduced, and instead the ability of cells to proliferate, migrate, and secrete various ECM and cytokines is acquired. VSMCs are a cell type with phenotypic transformation ability. In the diffuse intima thickening of preatherosclerotic lesions, VSMCs migrate from the middle membrane to the intima, and complete the transformation from the static "contraction" phenotype to the active "synthesis" phenotype [15]. "Synthetic" VSMCs are characterized by reduced expression of constrictor proteins, enhanced expression of growth factors, receptors, and ECM proteases, and migration and proliferation from the midmembrane to the intima, contributing to the formation of initial atherosclerotic plaques. In the later stages, they multiply and aggregate further, forming fibrous caps to stabilize fragile plaques. Earlier studies have shown that VSMCs can stabilize atherosclerotic plaques and protect them from rupture by promoting fibrous cap formation, which is thought to have a beneficial effect. VSMCs can transform into multiple phenotypes during atherosclerosis, including calcification (osteogenesis, chondrogenesis, and osteoclysis) and macrophage phenotypes. On the one hand, some VSMCs acquire macrophage properties during phenotypic transformation, differentiating into lipid-loaded foam cell-like macrophages that contribute to atherosclerosis by reducing their ability to clear lipids, dead cells, and necrotic debris and by increasing inflammation [16]. VSMCs, on the other hand, convert to calcified phenotypes. Phenotypic plasticity and osteochondral differentiation of VSMCs play a key role in atherosclerotic intima calcification [4]. Intima calcification is associated with atherosclerotic adverse events such as plaque rupture, myocardial infarction and stroke. VSMCs participate in the whole process of atherosclerosis by promoting cell proliferation by regulating phenotypes. Therefore, elucidating the mechanism of proliferation and migration of VSMCs provides a direction for the intervention of atherosclerosis.

Various miRNAs have been shown to be tightly involved in atherosclerosis, which played critical roles in promoting or inhibiting the proliferation and motion of VSMCs. For instance, Zhang et al. suggested that E2F1/SNHG7/miR-186-5p/MMP2 axis modulated the proliferation and migration of vascular endothelial cell in atherosclerosis [17]. Xie et al. found that LOXL1-AS1/miR-515-5p/STAT3 positive feedback loop facilitated cell proliferation and migration in atherosclerosis [18]. Wang et al. indicated that miR-377-3p inhibited atherosclerosis-associated vascular smooth muscle cell proliferation and migration through targeting neuropilin2 [19]. Liu et al. demonstrated that PRDM16 upregulation induced by miR-448 inhibition alleviates atherosclerosis via the TGF-beta signaling pathway inactivation [20]. It was indicated that miR-552 was tightly involved in the development of different kinds of cancers. For instance, Cai et al. revealed that miR-552-5p facilitated osteosarcoma cell proliferation and metastasis by targeting WIF1 [21]. Feng et al. found that upregulation of miR-552 promoted the proliferation, migration, and invasion of gastric cancer cells [22]. Cao et al. discovered that miR-552 promoted tumor cell proliferation and migration by directly targeting DACH1 by the Wnt/beta-catenin signaling pathway in colorectal cancer [23]. However, the biological function of miR-552 and its molecular mechanisms in AS remains unclear.

In this study, the expression of miR-552 was identified in HBVSMCs and mVSMCs using qRT-PCR and the results indicated that miR-552 was higher expressed in HBVSMCs treated with PDGF-bb compared to the non-treat group. Additionally, we verified that SKI was a direct target of miR-552 by miRWalk and dual-luciferase assay. The result illustrated that miR-552 reduced the SKI wild-type 3’-UTR activity in HBVSMCs. The result of **Fig 4** showed that SKI could reverse the promotional effect of miR-552 on the proliferation and migration of HBVSMCs and mVSMCs, suggesting that SKI blocked proliferation, invasion and migration of HBVSMCs and mVSMCs. In addition, Rashidian et al. suggested that SKI regulated Hippo and TAZ signaling to suppress breast cancer progression [24]. Xie et al. identified that SKI regulated Smads and TAZ signaling to suppress lung cancer progression [25], which was in agreement with the present research.

In recent years, plenty of TFs have been shown to be associated with atherosclerosis. For example, Erbilgin et al. suggested that TF Zhx2 deficiency reduced atherosclerosis and promoted macrophage apoptosis in mice [26]. Kotla et al. discovered that the TF CREB enhanced interleukin-17A production and inflammation in a mouse model of atherosclerosis [27].

Wang et al. revealed the contribution of TF EB to adipoRon-induced inhibition of arterial smooth muscle cell proliferation and migration [28]. In our study, JASPAR software online was used to predict TFs regulating promoters of miR-552 and the ATF4 were determined by qRT-PCR assay. Subsequently, we assessed the impact of ATF4 elevation on SKI expression via western blot analysis and miR-552 expression in accordance to qRT-PCR assay. As indicated in Fig **5D–5F**, the result exhibited that ATF4 upregulation could promote proliferation, invasion and migration of HBVSMCs and mVSMCs, which could be reversed by miR-552 decline. After that, the regulatory association between ATF4 and miR-552 was detected by ChIP assay and luciferase reporter assay. The result showed that ATF4 could transcriptionally modulate miR-552 expression.

There are still some limitations in the present study. First of all, we did not explore in detail what pathway or mechanism SKI promotes the proliferation of VSMCs. Second, although many articles have reported that smooth muscle proliferation causes atherosclerosis, we did not clarify this in animal models. Thirdly, whether other more important molecules play an important role in the proliferative effect of miR-522 needs further exploration.

## Conclusion

Taken together, our results indicated that ATF4-miR-552-SKI axis regulated the proliferation and migration of HBVSMCs and mVSMCs, which were closely associated with AS. Thus, our findings might offer a novel therapeutic method for the prevention and treatment of AS.

## Supporting information

S1 Table All special primers were enrolled in this study. (DOCX)

S1 Data (ZIP)
