# Supplementary material for: ATF4 promotes brain vascular smooth muscle cells proliferation, invasion and migration by targeting miR-552-SKI axis
Source: PLoS One. 2022 Jul 20;17(7):e0270880. doi: 10.1371/journal.pone.0270880 (PMC9299314; doi:10.1371/journal.pone.0270880)
Supplement: S1 Table — (DOCX) [file pone.0270880.s001.docx]

**Table S1**. All special primers were enrolled in this study.

| **Primer** | **Sequence** |
| --- | --- |
| SKI  BTF3L4 | Forward: 5’-GAGAAATTCGACTATGGCAACAAG-3’  Reverse: 5’-GGCCGAAATCAGATACTTTAAC-3′  Forward: 5’-CAGAAAGAAGAAGGTGGTA-3’  Reverse: 5’-TGAAATGAATAACTGTCCC-3’ |
| GAPDH  EDARADD    SGK1  SYNGR2  GBA2  NHLRC3  ATF4  SOX15    POU4F1  IKZF1  PRDM1  MiR-552 | Forward: 5'-TGACTTCAACAGCGACACCCA-3'  Reverse: 5'-CACCCTGTTGCTGTAGCCAAA-3'  Forward: 5'-GACCAACCCAAAGAGGACAG-3'  Reverse: 5'-CCAGAATGATGAGGCACCAT-3'  Forward: 5′- GCAGAAGAAGTGTTCTATGCAGT-3′  Reverse: 5′-CCGCTCCGACATAATATGCTT-3′  Forward: 5′-CCAACCAGTGGGCAGTCAC-3′  Reverse: 5′-GGGAAAGTCCAGGGCAGAG-3′’  Forward: 5′‐GTATGTGTTGTTTTTTTCAGGC‐3′  Reverse: 5′‐GCAATAACGGTTTTGTAGTGG‐3′  Forward: 5’-TTGTTCAAGTCTTGGGTAC-3’;  Reverse: 5’-AATCTGTTATTCAATCCTCC-3’  Forward: 5′-GGTTCTCCAGCGACA AGG-3′  Reverse: 5′-TCTCCAACATCCAATCTGTCC-3′  Forward: 5′-GAACAGGTTGGAAGCAAAGGC-3′  Reverse: 5′-GAACAGGTTGGAAGCAAAGGC-3′  Forward:5'-GAG​GCC​TAT​TTT​GCC​GTA​CA -3'  Reverse:5’-TTT​CAT​CCG​CTT​CTG​CTT​CT-3’  Forward: 5'-GAGTGACAGAGTCGTGGTTACAT-3'  Reverse: 5'-GCCCTTCTGGGTGAATGAG-3'  Forward: 5’-TAAAGCAACCGAGCACTGAGA-3’  Reverse: 5’-ACGGTAGAGGTCCTTTCCTTTG-3’  Forward: 5’- CCGCACAGGTGACTGGTTAGA-3’  Reverse: 5’- GTGCAGGGTCCGAGGT-3’ |
