# Supplementary figures and images for: ATF4 promotes brain vascular smooth muscle cells proliferation, invasion and migration by targeting miR-552-SKI axis
Source: PLoS One. 2022 Jul 20;17(7):e0270880. doi: 10.1371/journal.pone.0270880 (PMC9299314; doi:10.1371/journal.pone.0270880)

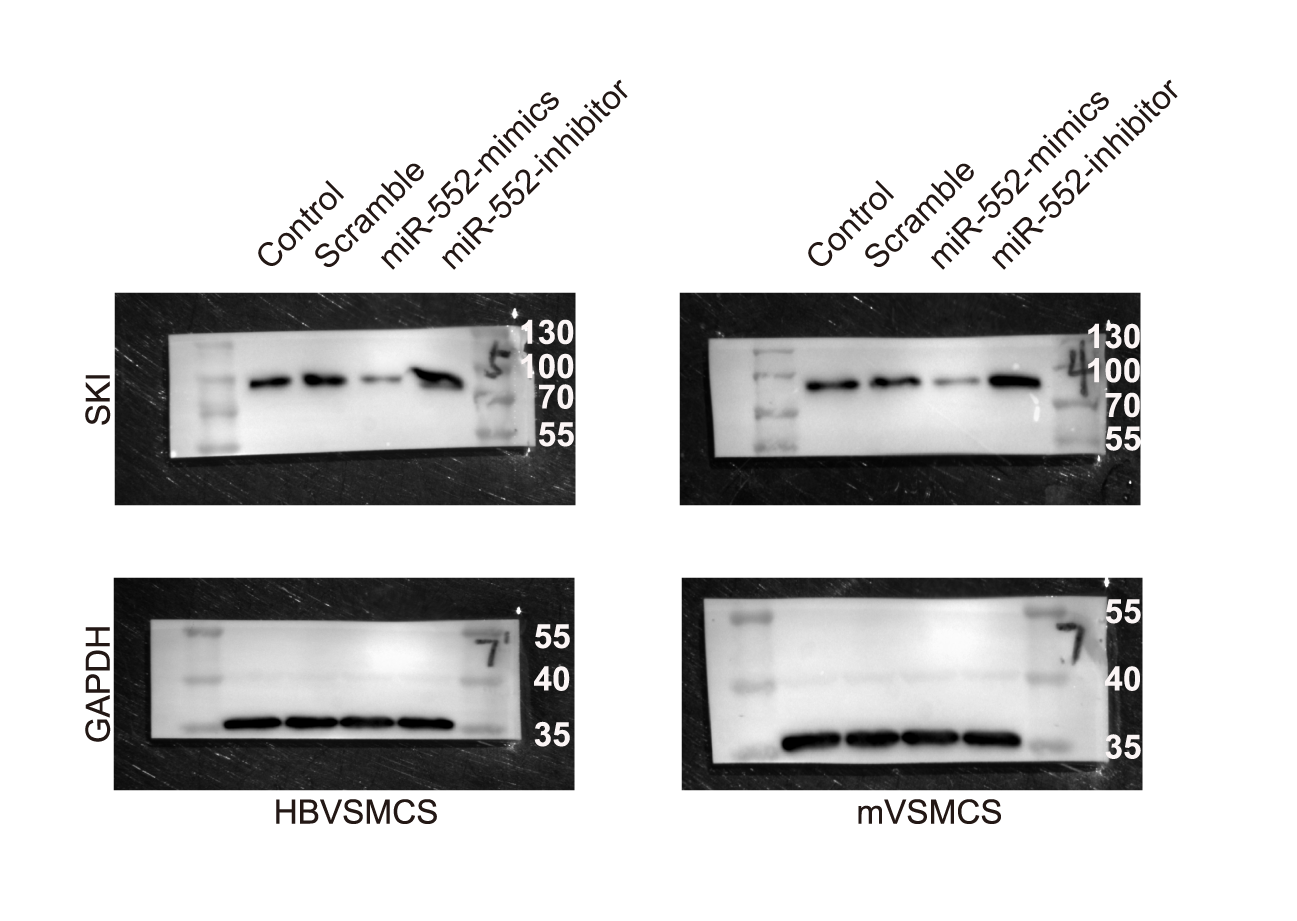

Supplement: S1 Data — (ZIP) [file pone.0270880.s002.zip › Fig. 3C.tif]

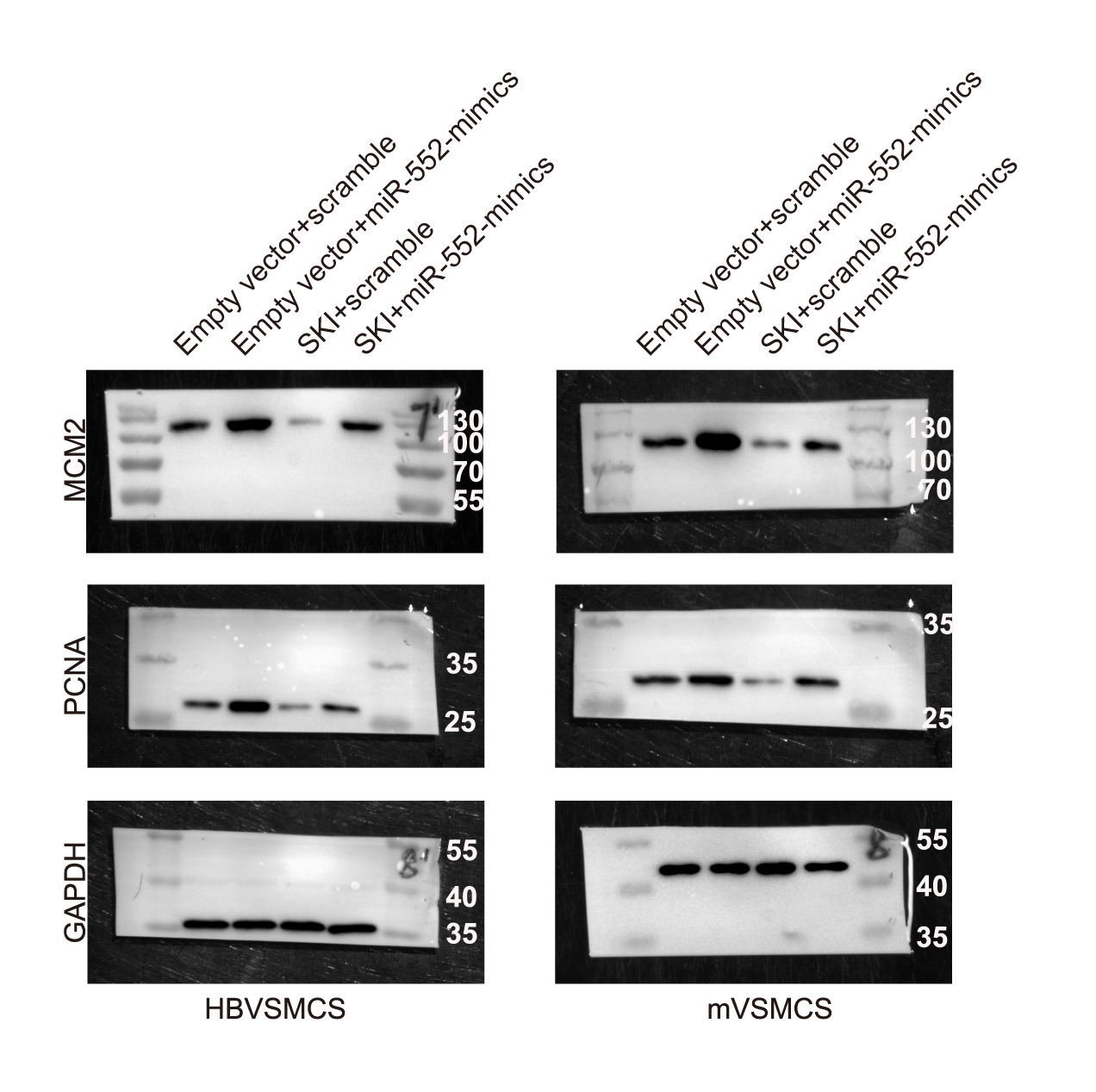

Supplement: S1 Data — (ZIP) [file pone.0270880.s002.zip › Fig. 4D.tif]

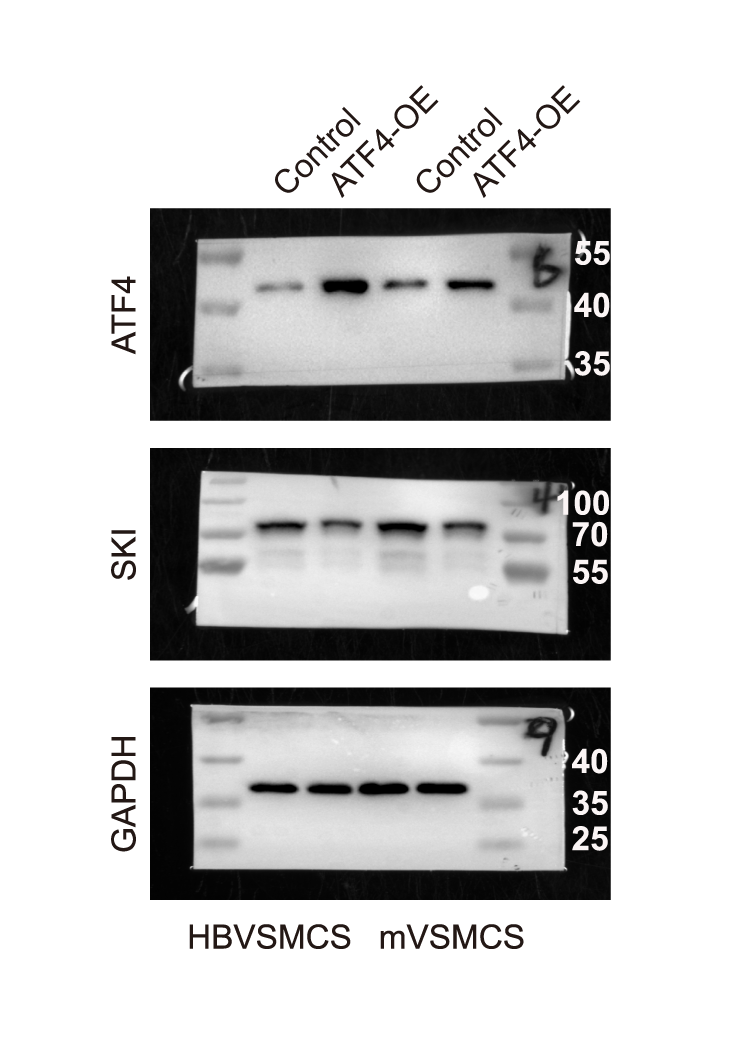

Supplement: S1 Data — (ZIP) [file pone.0270880.s002.zip › Fig. 5B.tif]

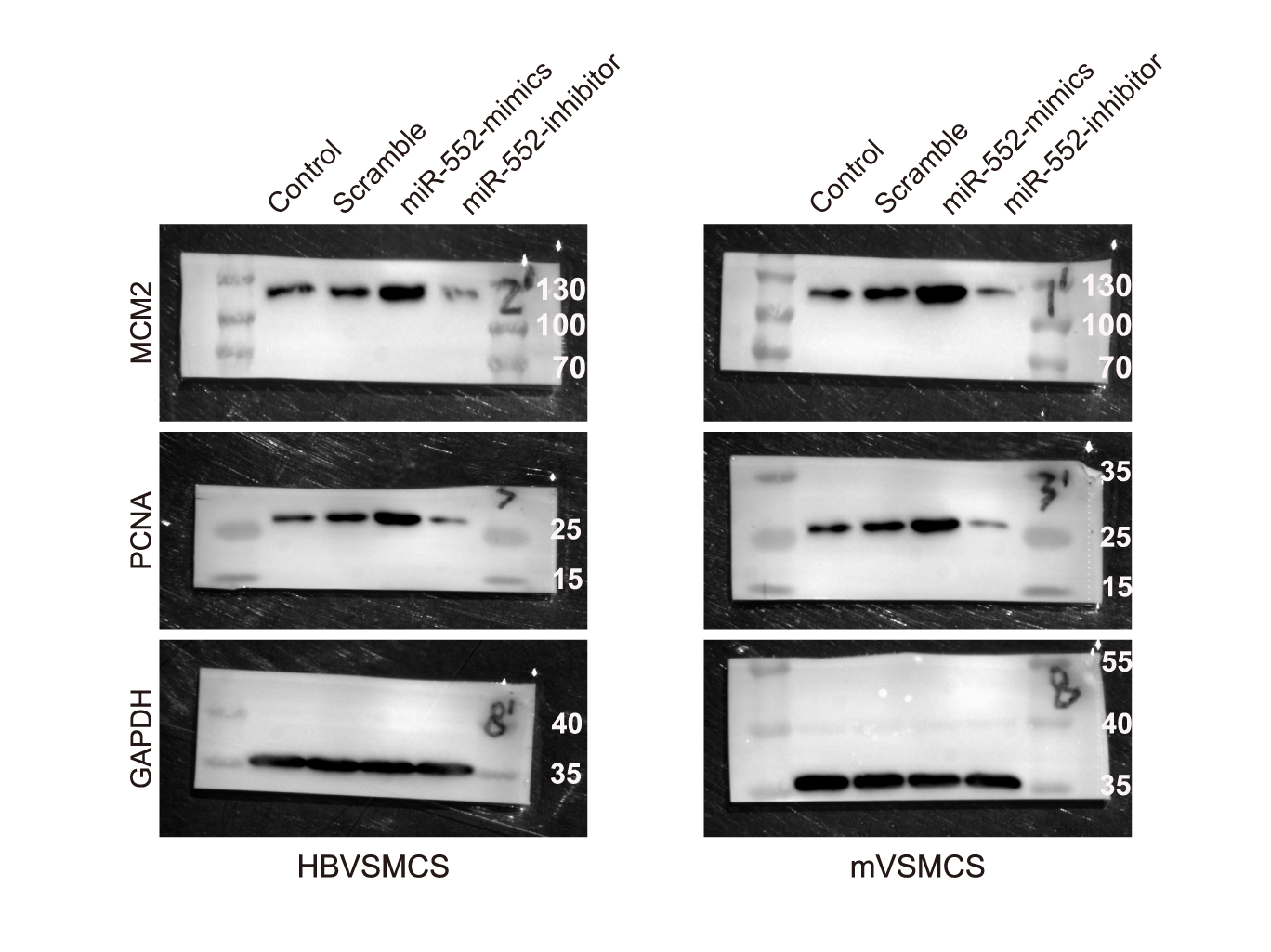

Supplement: S1 Data — (ZIP) [file pone.0270880.s002.zip › Fig. 2E.tif]
